# Síndrome de Adams-Oliver y complicaciones asociadas: reporte de una familia en Colombia y revisión de la literatura

**DOI:** 10.7705/biomedica.6524

**Published:** 2022-12-01

**Authors:** Olga Lucía Morales, Jerly Maybelline Díaz, Jorge Hernán Montoya

**Affiliations:** 1 Departamento de Pediatría, Grupo de Investigación Pediaciencias, Universidad de Antioquia, Hospital San Vicente Fundación, Clínica Noel, Medellín, Colombia Universidad de Antioquia Universidad de Antioquia Medellín Colombia; 2 Universidad Militar Nueva Granada, Bogotá, D.C., Colombia Universidad Militar Nueva Granada Universidad Militar Nueva Granada Bogotá D.C Colombia; 3 Hospital San Vicente Fundación, Medellín, Colombia Hospital San Vicente Fundación Medellín Colombia

**Keywords:** síndrome de Adams-Oliver, displasia ectodérmica, deformidades congénitas de las extremidades, patrón de herencia, Adams-Oliver syndrome, ectodermal dysplasia, limb deformities, congenital, inheritance pattern

## Abstract

El síndrome de Adams-Oliver es un trastorno congénito raro, caracterizado por aplasia cutis congénita en el cuero cabelludo, defectos terminales transversales de las extremidades y piel *marmorata* telangiectásica congénita. Este puede presentarse debido a diferentes patrones de herencia de tipo autosómico dominante o autosómico recesivo, o por mutaciones dominantes *de novo*.

Aunque el síndrome de Adams-Oliver es una enfermedad poco frecuente, es importante conocer sus características clínicas y patrones de herencia, para así establecer un correcto diagnóstico y sus posibles complicaciones durante el seguimiento.

En el presente estudio, se describe el caso de una adolescente con síndrome de Adams-Oliver con patrón de herencia autosómica dominante, hipertensión pulmonar y bronquitis plástica. Había varios miembros de su familia con el mismo compromiso

## Abstract (en)

The Adams-Oliver syndrome is a rare congenital disorder characterized by aplasia cutis congenita of the scalp, terminal transverse limb defects, and congenital telangiectatic cutis marmorata. It can occur through different inheritance patterns: autosomal dominant, autosomal recessive, or *de novo* dominant mutations.

Although the Adams-Oliver syndrome is a rare disease, it is essential to know its clinical characteristics and inheritance patterns, to establish a correct diagnosis and its possible complications during follow-up.

In the present study, we describe the case of an adolescent with Adams-Oliver syndrome with an autosomal dominant inheritance pattern, pulmonary hypertension and plastic bronchitis, and several compromised family members.

Forrest H. Adams y C. P. Oliver describieron este síndrome por primera vez en 1945 en ocho miembros afectados de tres generaciones de una misma familia ^1^. Desde entonces, se han reportado más de 200 casos de pacientes con esta misma entidad, y se estima una incidencia de 0,44 por 100.000 nacidos vivos. El síndrome de Adams-Oliver se caracteriza por aplasia cutis congénita del cuero cabelludo y defectos terminales transversales de las extremidades. Asimismo, existe un espectro amplio de afectaciones con compromiso cutáneo, neurológico, cardiovascular, locomotor, renal y ocular ^2^.

Esta enfermedad se ha asociado con diversos patrones de herencia, los cuales dependen de los genes implicados. Las mutaciones en los genes *ARHGAP31*^3^, *NOTCH1*^4^^,^^5^, *DLL4*^6^ o *RBPJ*^7^ se han correlacionado con la herencia autosómica dominante, mientras que las mutaciones en los genes *DOCK6*^8^ o *EOGT*^9^ se asocian con la herencia autosómica recesiva. Asimismo, en algunos estudios se ha evidenciado una fuerte correlación entre el genotipo y la expresión del fenotipo, como el hallazgo de una proporción significativamente mayor de anomalías cardíacas congénitas en pacientes con *AOS5* -una variante del gen *NOTCH1* - lo que podría representar un subtipo distinto de síndrome de Adams-Oliver asociado con malformaciones cardíacas ^5^. De igual manera, la aparición aislada de este síndrome en algunas familias sugiere mutaciones dominantes *de novo*^4^^,^^10^.

Teniendo en cuenta la poca frecuencia de este síndrome, así como su relación familiar, se describe el caso de una adolescente y su familia, para que se tenga en cuenta como sospecha diagnóstica de nuevos casos.

## Caso clínico

Se trata de una paciente de sexo femenino de 13 años, producto del tercer embarazo, a término, cuya talla al nacer fue de 47 cm y cuyo peso fue de 2.470 g. Se practicó una cesárea debido a sufrimiento fetal agudo y sangrado genital masivo posterior a la amniotomía, que laceró estructuras varicosas del cuero cabelludo de la paciente.

**Figura 1:**
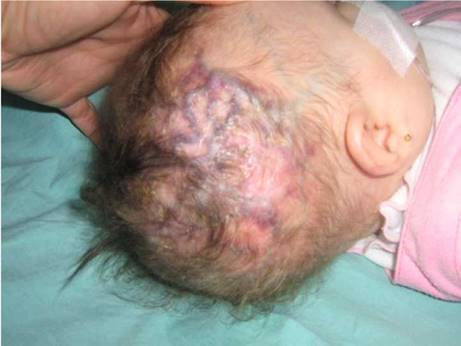
Paciente con síndrome de Adams-Oliver con dilatación y tortuosidad venosa en el cuero cabelludo

**Figura 2:**
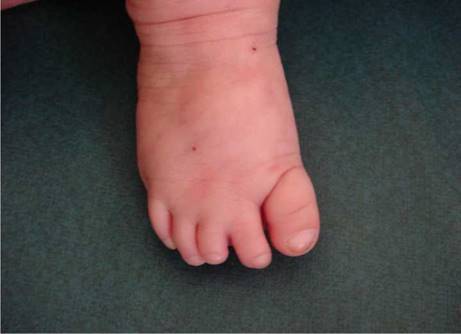
*Cutis marmorata* telangiectásica y defectos transversales en los dedos de los pies. En el pie derecho se observa sindactilia entre el segundo y el tercer dedo, hipoplasia del tercero y uñas displásicas, con un pliegue cutáneo profundo que rodea el primer dedo o dedo gordo.

Al nacimiento, fue hospitalizada por choque hipovolémico, y se evidenció aplasia cutis congénita, vasos prominentes y tortuosos en el cuero cabelludo (figura 1), defectos transversales en los dedos de los pies, hipoplásicos, presencia de sindactilia y *cutis marmorata* telangiectásica (figura 2). En la angiorresonancia cerebral, no se observaron lesiones vasculares intracraneanas.

A los dos meses de vida, presentó episodios de apnea y cianosis con recuperación espontánea, con resultados normales en la ecocardiografía y demás estudios clínicos. A los cuatro meses, presentó paro respiratorio con recuperación rápida tras el inicio de oxígeno suplementario y presión positiva. Una nueva ecocardiografía reportó signos indirectos de hipertensión pulmonar. Los resultados de la angiorresonancia de tórax y abdomen, y la polisomnografía, fueron normales.

A los 3 años consultó por tos de dos días de evolución, con expectoración de moldes de fibrina hemoptoicos, y se diagnosticó bronquitis plástica. Este diagnóstico se confirmó por medio de estudios de histopatología, en los cuales se observaron moldes de tejido con presencia de fibrina y algunos linfocitos acompañados de células espumosas. Se inició tratamiento con dornasa alfa, N-acetilcisteína nebulizada y terapia respiratoria. Hasta el presente, la paciente continúa presentando periodos con expectoración de membranas.

A los 3 años, el resultado de la ecocardiografía reportó hipertensión pulmonar moderada y dilatación de cavidades derechas, por lo que se inició su manejo con sildenafilo. En los controles ecocardiográficos, se reportó una diminución del grado de hipertensión pulmonar, de leve hasta nula.

A los 5 años, presentó fatiga y cianosis cuando realizaba alguna actividad física. En el ecocardiograma se encontró una hipertensión pulmonar moderada; en el electrocardiograma, hipertrofia ventricular derecha, y en el cateterismo cardíaco, una hipertensión pulmonar moderada con reactividad vascular al oxígeno. Por estos hallazgos, se continuó el tratamiento con sildenafilo y oxígeno domiciliario. Posteriormente, en el mismo año, en el ecocardiograma de control se encontraron signos indirectos de hipertensión pulmonar y la caminata de 6 minutos fue normal.

A los 7 años, se observaron várices y edema de miembros inferiores, descartándose trombosis venosa profunda.

A la misma edad, y posteriormente a los 13 años, los resultados del ecocardiograma de control informaron signos indirectos de hipertensión pulmonar leve y la caminata de 6 minutos fue normal. En la actualidad, el seguimiento ambulatorio y la continuidad del suministro de los medicamentos se han visto afectados por dificultades socioeconómicas e inconvenientes administrativos por parte de su aseguradora.

**Figura 3:**
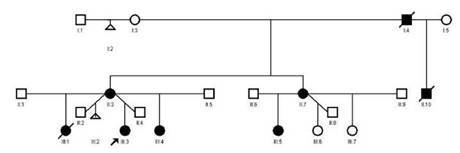
Árbol genealógico con patrón de herencia autosómica dominante

Entre los antecedentes familiares (figura 3), el abuelo materno presentaba una alteración vascular en el cuero cabelludo y vasos prominentes en varias partes del cuerpo; la tía materna tenía defectos transversales de los dedos de los pies, malformaciones vasculares en el cuero cabelludo y *cutis marmorata* telangiectásica.

La madre presentaba lesiones similares en el cuero cabelludo y, en ese momento, una cicatriz alopécica en el vértice de la cabeza, anormalidades de los dedos, venas prominentes en varias partes del cuerpo, con antecedente de retardo en el crecimiento intrauterino. En la actualidad, manifiesta cansancio, con limitación para realizar actividades de mediana intensidad, pero, por dificultades socioeconómicas, no tiene seguimiento médico ni ecocardiografía actualizada.

La hermana mayor murió a los 4 meses de edad, a causa de una miocarditis aguda (según el informe de patología). Sin embargo, presentaba lesiones vasculares en el cuero cabelludo y la frente, y piel *marmorata*, sin alteraciones en los dedos. Por otro lado, la hermana menor presentaba malformaciones del cuero cabelludo, sin cambios en la piel o en los dedos.

El presente estudio es una investigación de bajo riesgo, según la definición del Invima (Resolución 8430 de 1993) y los principios de la declaración de Helsinki. Se obtuvo la aceptación para la publicación de la historia clínica y las imágenes, junto con el consentimiento informado por parte de la madre de la paciente. Además, se obtuvo aprobación por parte del Comité de Ética del Hospital San Vicente Fundación.

## Discusión

En el presente estudio, se describe el caso de una paciente con síndrome de Adams-Oliver, con las características clínicas clásicas de esta entidad: aplasia cutis congénita, que se presenta en el 80 % de los casos, defectos terminales transversales de las extremidades, en el 85 %, y *cutis marmorata* telangiectásica, en 20 al 25 % de los casos ^11^. Entre los hallazgos que se destacan, se encuentra la hipertensión pulmonar, reportada en el 5 % de los casos, además del retardo del crecimiento intrauterino y la bronquitis plástica, no descrita en este grupo de pacientes en la literatura científica.

El síndrome de Adams-Oliver es una enfermedad rara, con amplia variedad de manifestaciones clínicas, suficientes para establecer el diagnóstico sin requerir estudios genéticos, como se evidencia en el presente caso (cuadro 1). Sin embargo, se han descrito algunos genes relacionados con hallazgos clínicos específicos (cuadro 2).

**Cuadro 1:** Criterios diagnósticos del síndrome de Adams-Oliver ^2^

1. Hallazgos sugestivos de aplasia cutis congénita del cuero cabelludo y defectos terminales transversales de las extremidades 2. Aplasia cutis congénita del cuero cabelludo y defectos terminales transversales de las extremidades, y un familiar en primer grado con hallazgos consistentes con síndrome de Adams-Oliver 3. Aplasia cutis congénita del cuero cabelludo y defectos terminales transversales de las extremidades, y una variante patogénica de un gen relacionado con síndrome de Adams-Oliver autosómico dominante (*ARHGAP31, DLL4, NOTCH1 o RBPJ*), o dos variantes patogénicas de un gen relacionado con el síndrome de Adams-Oliver autosómico recesivo (*DOCK6* o *EOGT*)

Reproducido con autorización de: Lehman A, Wuyts W, Patel MS. Adams-Oliver Syndrome. In: Adam MP, Everman DB, Mirzaa GM, *et al*., editors. GeneReviews® [Internet]. Seattle (WA): University of Washington, Seattle; 1993-2022. Disponible en: https://www.ncbi.nlm.nih.gov/books/NBK355754/

**Cuadro 2:** Manifestaciones clínicas del síndrome de Adams-Oliver y mutaciones genéticas asociadas

Órgano o sistema	Afectación	Mutación genética asociada
Cutáneo o craneal	Cutis marmorata telangiectásica	DLL4 ^11^, AOS3, AOS4 ^3^
	Aplasia cutis congénita	
Ocular	Vascularización retiniana incompleta o anormal	DOCK6 ^13^, AOS2, AOS6 ^4^
	Sangrado retiniano	
	Desprendimiento de retina	
	Retinopatía proliferativa ^12^	
	Microftalmos	
	Cataratas	
	Esotropía	
Neurológico	Calcificaciones intracraneales	*DOCK6* (alto riesgo), *ARHGAP31* (bajo riesgo), *AOS2* (mal pronóstico) ^4^
	Discapacidad intelectual	Raro: *NOTCH1*, *DLL4 y EOGT*^14^
	Convulsiones	
	Parálisis cerebral	
	Trastornos del espectro autista	
	Microcefalia	
	Displasia cortical	
	Mielinización retardada	
Cardiovascular	Malformaciones cardiacas ^15^	*NOTCH1*, ^14^*DOCK6*, *DLL4 y EOGT* (alto riesgo), *RBPJ y RHGAP31* (bajo riesgo), *AOS5*^16^
	Estenosis de la vena pulmonar	
	Hipertensión pulmonar	
	Irrigación sanguínea intestinal deficiente	
	Vasos aberrantes de vellosidades coriónicas placentarias	
	Hipertensión portal idiopática	
	Venas del cuero cabelludo tortuosas y dilatadas ^2^	
Locomotor	Defectos terminales transversales de las extremidades	*ARHGAP31, AOS3, DOCK6 y EOGT*^14^
	Dedos hipoplásicos	
	Braquisindactilia	
	Ectrodactilia ^13^	
Otros	Paladar hendido	
	Gastrosquisis	
	Malformaciones renales	
	Pezones supernumerarios	
	Retraso del crecimiento intrauterino ^11^	

La hipertensión pulmonar es poco frecuente en este síndrome, pero se relaciona con gran morbimortalidad ^2^. Varios autores han propuesto una vasculopatía como causa de esta condición, incluyendo diferentes hipótesis como trombosis ^17^, interrupción vascular de cualquier etiología ^18^, hipoplasia arterial ^19^^,^^20^, anomalías específicas de las células endoteliales ^21^ y reclutamiento anormal de los pericitos en los vasos sanguíneos, lo que podría explicar los vasos dilatados y tortuosos, así como los defectos terminales de las extremidades ^22^.

La bronquitis plástica es una complicación rara y grave de las enfermedades respiratorias asociadas con trastornos bronquiales hipersecretores difusos, anomalías linfáticas e infecciones, después de cirugías para cardiopatías congénitas, principalmente la de Fontan. Se caracteriza por la formación de moldes de cilindros bronquiales espesos que conducen a la oclusión total o parcial de la vía aérea, los cuales pueden ser expectorados. Se clasifican en dos tipos: en el tipo 1, más frecuente con enfermedades pulmonares, los moldes están formados por células inflamatorias, y en el tipo 2, relacionado con cardiopatías, los moldes son hipocelulares y contienen mucina ^23^.

Nuestra paciente presenta expectoración recurrente de dichos moldes, los que consideramos que son más probablemente del tipo 2 por el escaso número de células y podrían atribuirse a las alteraciones del endotelio con anormalidades vasculares y linfáticas que pueden generar hipertensión venosa central, estasis linfática y salida de linfa al árbol bronquial, con pérdida de células linfoides e inmunoglobulinas, lo que implica mayor propensión a infecciones, como se describe en el presente caso ^23^. Este es el primer caso descrito de bronquitis plástica asociada con el síndrome de Adams-Oliver, según la bibliografía consultada.

Por otro lado, como se mencionó anteriormente, hay diferentes formas de herencia involucradas en este síndrome. En el presente caso, al observarse afectación de ambos sexos, sin saltos generacionales, se puede concluir que se trata de un patrón de herencia autosómica dominante, como en la mayoría de los reportes de casos ^24^. Asimismo, se han involucrado diferentes mecanismos fisiopatológicos con este síndrome, como los defectos del citoesqueleto de actina por los genes *ARHGAP31 y DOCK6*, la alteración de la señalización de Notch por el *RBPJ y* el *NOTCH1*, y la alteración postraduccional de la O-acetilación por el *EOGT*^14^.

De igual manera, se han propuesto otros posibles mecanismos fisiopatológicos, entre los que se encuentran la irrigación vascular anormal en la fase de embriogénesis y numerosos microtrombos en la placenta, alteración del suministro de sangre en las ramas de las arterias subclavia y vertebral, detención del desarrollo en la formación de tejidos blandos y esqueléticos, y bandas amnióticas ^24^.

Entre los diagnósticos diferenciales del síndrome de Adams-Oliver, se pueden encontrar el síndrome cuero cabelludo-oreja-pezón, la aplasia cutis congénita aislada ^2^, la secuencia de bandas amnióticas ^25^, el síndrome de Poland ^2^ y el síndrome de Goltz ^25^.

## Conclusiones

Aunque el síndrome de Adams-Oliver es una enfermedad poco frecuente, es importante conocer su existencia, sus características clínicas y sus patrones de herencia, para así establecer su correcto diagnóstico y posibles complicaciones durante el seguimiento. En el presente caso, la paciente presentó hipertensión pulmonar y bronquitis plástica; esta última no había sido descrita anteriormente en esta entidad. Se requieren más estudios para establecer el pronóstico de estos pacientes a corto, mediano y largo plazo.
